# WHO criteria for diabetes in pregnancy: a retrospective cohort

**DOI:** 10.1186/s12884-022-04708-w

**Published:** 2022-05-03

**Authors:** Tatiana A. Zaccara, Cristiane F. Paganoti, Fernanda C. F. Mikami, Rossana P. V. Francisco, Rafaela A. Costa

**Affiliations:** 1 Departamento de Obstetricia e Ginecologia da Faculdade de Medicina da, Universidade de São Paulo, Sao Paulo, SP Brazil; 2 Divisão de Clinica Obstetrica do Hospital das Clínicas da Faculdade de Medicina da, Universidade de Sao Paulo, Sao Paulo, SP Brazil

**Keywords:** Diabetes in pregnancy, Gestational diabetes mellitus, World Health Organization, Glucose Tolerance Test

## Abstract

**Background:**

Recognizing that hyperglycemia in pregnancy can impact both individually a patient’s health and collectively the healthcare system and that different levels of hyperglycemia incur different consequences, we aimed to evaluate the differences and similarities between patients who met the diagnostic criteria for gestational diabetes mellitus (GDM) or diabetes in pregnancy (DIP) according to the World Health Organization diagnostic criteria based on the 75 g oral glucose tolerance test (OGTT).

**Methods:**

This retrospective study included a cohort of 1064 women followed-up at the Gestational Diabetes Unit of Hospital das Clinicas da Faculdade de Medicina da Universidade de Sao Paulo (Sao Paulo, Brazil). Patients were classified into GDM and DIP groups, according to their OGTT results. Their electronic charts were reviewed to obtain clinical and laboratory data for all participants.

**Results:**

Women in the DIP group had a higher pre-pregnancy body mass index (30.5 vs 28.1 kg/m^2^, odds ratio [OR] 1.07, 95% confidence interval [CI] 1.02–1.11), more frequently experienced GDM in a previous pregnancy (25% vs. 11%, OR 2.71, 95% CI 1.17–6.27), and were more likely to have chronic hypertension (43.1% vs. 23.5%, OR 2.46, 95% CI 1.47–4.11), a current twin pregnancy (10.8% vs. 2.9%, OR 4.04, 95% CI 1.70–9.61), or require insulin (46.1% vs. 14.3%, OR 5.14, 95% CI 3.06–8.65) than those in the GDM group. Patients in the DIP group also had a higher frequency of large-for-gestational-age infants (12.3% vs. 5.1%, OR 2.78, 95% CI 1.23–6.27) and abnormal postpartum OGTT (45.9% vs. 12.6%, OR 5.91, 95% CI 2.93–11.90) than those in the GDM group. Nevertheless, in more than half of the DIP patients, glucose levels returned to normal after birth.

**Conclusions:**

Diabetes in pregnancy is associated with an increased risk of adverse perinatal outcomes but does not equate to a diagnosis of diabetes post-pregnancy. It is necessary to identify and monitor these women more closely during and after pregnancy. Keeping patients with hyperglycemia in pregnancy engaged in healthcare is essential for accurate diagnosis and prevention of complications related to abnormal glucose metabolism.

## Background

Hyperglycemia is one of the most common complications in pregnancy, affecting an estimated 15.8% of live births in 2019 [1]. Of these, 83.6% were due to gestational diabetes mellitus (GDM), and 8.5% were due to diabetes first recognized during pregnancy [1]. All forms of hyperglycemia in pregnancy are associated with worse perinatal outcomes, but there are a few complications and different ways of management that are particular to each type of diabetes [2, 3].

In 2013, the World Health Organization (WHO) adopted the GDM criteria proposed by the International Association of Diabetes and Pregnancy Study Groups (IADPSG) (fasting plasma glucose [FPG] ≥ 5.1 mmol/L, and/or 1-h plasma glucose [1 h-PG] ≥ 10.0 mmol/L, and/or 2-h plasma glucose [2 h-PG] ≥ 8.6 mmol/L in the 75 g oral glucose tolerance test [OGTT]), with the caveat that the diagnostic criteria for non-pregnant adults should also apply to pregnant women [4]. According to this recommendation, patients with FPG ≥ 7 mmol/L at any time during pregnancy and/or 2 h-PG ≥ 11.1 mmol/L in the OGTT are diagnosed with diabetes in pregnancy (DIP), rather than with GDM. By definition, these pregnancies are associated with a greater risk of congenital malformations and diabetes-related complications, and these women would not require repeat testing postpartum to confirm a diagnosis of diabetes. Nevertheless, in our clinic, patients with abnormal OGTT are considered to have GDM, rather than DIP, irrespective of their glucose levels.

Studies on this population are scarce. This study compared the characteristics and outcomes of women with GDM or DIP, classified according to the OGTT levels, as proposed by the WHO.

## Methods

This was a single-center, cohort study of women from the Gestational Diabetes Unit in the Obstetrics Department of Hospital das Clinicas da Faculdade de Medicina da Universidade de Sao Paulo (Sao Paulo – Brazil), a tertiary hospital and reference center for high-risk pregnancies and fetal medicine. We analyzed the medical records of all patients, regardless of the number of fetuses, who were seen between 04/01/2012 and 03/26/2020 and diagnosed with GDM based on abnormal OGTT glucose levels (as defined by the IADPSG criteria). We aimed to evaluate the differences and similarities between patients who met the diagnostic criteria for gestational diabetes mellitus (GDM) or diabetes in pregnancy (DIP) according to the World Health Organization diagnostic criteria based on the 75 g oral glucose tolerance test (OGTT). Patients with diagnosis of type 1 or type 2 diabetes were not included in this study.

Pregnant women who visit our Obstetrics Department, are offered an FPG test at the first appointment. If the FPG level is ≥5.1 mmol/L and < 7 mmol/L, the patient is diagnosed with GDM and is followed up in the Gestational Diabetes Unit. If the FPG level is ≥7 mmol/L, the patient is diagnosed with overt diabetes and is followed up in the Antenatal Care Unit for Pregnant Women with Type 1 and Type 2 Diabetes. Universal screening with an OGTT is offered between 24 and 28 weeks to all patients who have an FPG level < 5.1 mmol/L at the first appointment and the thresholds proposed by the IADPSG are used to identify GDM cases (FPG ≥ 5.1 mmol/L, and/or 1 h-PG ≥ 10.0 mmol/L, and/or 2 h-PG ≥ 8.6 mmol/L). Even if, in this test, FPG is ≥7 mmol/L or 2 h-PG is ≥11.1 mmol/L, these patients are managed as GDM and are attended to in the corresponding unit.

All patients diagnosed with GDM in this study were followed up by a multidisciplinary antenatal team consisting of doctors, nurses, and dietitians. The patients received lifestyle change and nutritional recommendations and were also instructed to perform self-monitoring blood glucose measurements at least four times/day. If adequate glycemic control was not achieved with diet and exercise, insulin was used as the first option for pharmacological treatment. In our clinic, we follow patients with GDM until the gestational age of 39–40 weeks, when we offer labor induction or elective cesarean section, according to the fetus’ and patient’s health conditions and obstetric history. We do not perform elective cesarean section based only on the GDM diagnosis.

All patients diagnosed with GDM during pregnancy were offered an OGTT 6–12 weeks after delivery, and the American Diabetes Association criteria [3] were used to identify abnormal results, which included impaired fasting glucose (IFG), impaired glucose tolerance (IGT), and diabetes mellitus (DM). Impaired fasting glucose was defined as FPG levels of 5.6–6.9 mmol/L and IGT was defined as 2 h-PG levels of 7.8–11 mmol/L. Diabetes mellitus was diagnosed at FPG ≥ 7 mmol/L or 2 h-PG ≥ 11.1 mmol/L after a 75-g glucose load.

Electronic charts were reviewed, and the following data were obtained for each participant: age, pre-pregnancy body mass index (BMI), parity, history of GDM, family history of DM, connective tissue disease, chronic hypertension, asthma, smoking habit, history of polycystic ovary syndrome (PCOS), multiple pregnancy, FPG at the first appointment, gestational age at the screening OGTT, glucose levels on the OGTT, insulin requirements during pregnancy, as well as the fetus’ gestational age at birth, sex, weight, congenital malformations, stillbirth, and 5-minute Apgar score. The return rate for follow-up appointments and postpartum OGTT glucose levels were also recorded.

The study was conducted with the approval of the Ethics Committee of Hospital das Clinicas – FMUSP, Sao Paulo, Brazil (CAAE: 48868915.9.0000.0068). Informed consent was waived because of its retrospective nature. All methods were carried out in accordance with relevant guidelines and regulations.

### Statistical analysis

Patients were classified into two groups according to the results of the OGTT, based on the WHO proposal [4]: the GDM group (FPG 5.1–6.9 mmol/L, and/or 1 h-PG ≥ 10 mmol/L, and/or 2 h-PG 8.6–11 mmol/L) and the DIP group (FPG ≥ 7 mmol/L and/or 2 h-PG ≥ 11.1 mmol/L). Clinical and laboratory data were compared between the two groups. Data were presented as medians (interquartile ranges), or absolute numbers and percentages, as appropriate.

For quantitative variables, the Kolmogorov–Smirnov test was used to assess the normality of the data distribution. As the data did not have a normal distribution, the Mann–Whitney U test was used to compare the groups. Association analysis for categorical variables was performed using the chi-squared test or Fisher’s exact test, as applicable.

Since the persistence of hyperglycemia is a particular concern in cases with GDM and because patients with DIP could be permanently labeled as diabetic, we further classified patients according to the results of the puerperal OGTT, into normal (FPG ≤ 5.5 mmol/L and 2 h-PG ≤ 7.7 mmol/L) and abnormal (FPG ≥ 5.6 mmol/L or 2 h-PG ≥ 7.8 mmol/L) groups, and compared clinical and laboratory data, including the diagnosis of GDM or DIP, as dependent variables.

Univariate logistic regression was used to estimate the odds ratios (OR) and their respective 95% confidence intervals (CI) for comparison between the GDM and DIP groups and between normal and abnormal puerperal OGTT groups.

A multivariable logistic regression analysis was performed, which included statistically significant variables from the univariate logistic regression analysis and clinically relevant variables, to identify independent factors associated with the persistence of hyperglycemia after delivery (normal vs. abnormal puerperal OGTT groups). A log-linear function was used to estimate the probability of an abnormal postpartum OGTT (including IFG, IGT, and DM).

Statistical software SPSS version 26 (IBM SPSS Inc., Armonk, NY, USA) was used for statistical analysis, and a *P*-value < 0.05, was considered statistically significant.

## Results

Overall, 2150 patients were followed-up at the gestational diabetes unit during the aforementioned period. Of these, 1065 had abnormal FPG levels early in pregnancy and 1085 had abnormal OGTT results. Twenty-one patients were excluded because they did not complete the OGTT and therefore could not be classified as having DIP or GDM. The remaining 1064 patients were included in this study and were classified into two groups according to their OGTT glucose levels: 999 in the GDM group and 65 in the DIP group. In five patients of the DIP group, only the FPG value was above the threshold; in 54, only the 2 h-PG value exceeded the threshold, and in six, both values were above the thresholds.

The baseline, birth, and newborn characteristics of each group are presented in Table 1. Women in the DIP group had higher pre-pregnancy BMI values, a more frequent history of GDM in a previous pregnancy, and were more likely to have chronic hypertension, a current twin pregnancy, and require insulin. There were no statistically significant differences in terms of a family history of diabetes, asthma, or history of PCOS.

**Table 1:** Baseline, birth, and newborn characteristics of women followed up in the Gestational Diabetes Unit

Variable (n of recorded data)	GDM (*n* = 999) Median (IQR) N (%)	DIP (*n* = 65) Median (IQR) N (%)	Odds ratio (95% CI)
Age, years (*n* = 1064)	33 (29–37)	33 (28–37)	1.00 (0.96–1.04)
BMI, kg/m^2^ (*n* = 1042)	28.1 (23.8–31.3)	30.5 (26.2–33.9)	**1.07 (1.02–1.11)**
Nullipara at first appointment (*n* = 1064)	394/999 (39.4%)	33/65 (50.8%)	1.58 (0.96–2.61)
Family history of DM (*n* = 1064)	542/999 (54.3%)	35/65 (53.8%)	0.98 (0.60–1.63)
Previous GDM (*n* = 637) (for non-nullipara patients)	66/604 (10.9%)	8/32 (25.0%)	**2.72 (1.18–6.29)**
Connective tissue disease (*n* = 1043)	39/978 (4.0%)	2/65 (3.1%)	0.76 (0.18–3.24)
Chronic hypertension (*n* = 1064)	235/999 (23.5%)	28/65 (43.1%)	**2.46 (1.47–4.11)**
Asthma (*n* = 865)	42/800 (5.3%)	4/65 (6.2%)	1.18 (0.41–3.41)
Smoking habit (*n* = 1057)	61/992 (6.1%)	5/65 (7.7%)	1.27 (0.49–3.28)
Twin pregnancy (*n* = 1064)	29/999 (2.9%)	7/65 (10.8%)	**4.04 (1.70–9.61)**
History of PCOS (*n* = 1002)	77/937 (8.2%)	5/65 (7.7%)	0.93 (0.36–2.39)
Fasting glucose at first appointment (mmol/L) (*n* = 907)	4.5 (4.2–4.7)	4.6 (4.2–4.8)	1.02 (0.98–1.06)
Gestational age at OGTT (weeks) (*n* = 1064)	26 + 4 ([25 + 0] – [28 + 3])	27 + 1 ([25 + 4] – [28 + 4])	1.00 (0.99–1.01)
Fasting plasma glucose-OGTT (mmol/L) (*n* = 1064)	5.0 (4.6–5.3)	5.4 (5.0–6.3)	**1.09 (1.06–1.11)**
1-h plasma glucose (mmol/L) (*n* = 1044)	9.0 (7.9–10.2)	11.5 (10.5–12.8)	**1.05 (1.04–1.06)**
2-h plasma glucose (mmol/L) (*n* = 1064)	8.6 (7.3–9.3)	11.9 (11.3–13.0)	**1.12 (1.09–1.14)**
Insulin use during pregnancy (*n* = 1045)	140/980 (14.3%)	30/65 (46.1%)	**5.14 (3.06–8.65)**
Gestational age at birth (weeks + days) (*n* = 854)	38 + 2 ([37 + 1] – [39 + 3])	37 + 1 ([36 + 0] – [38 + 6])	0.98 (0.97–1.00)
Cesarean section (*n* = 865)	509/804 (63.3%)	49/61 (80.3%)	**2.37 (1.24–4.52)**
Newborn sex = female (*n* = 893)^a^	416/828 (50.2%)	30/65 (46.2%)	1.18 (0.11–6.75)
Congenital malformation (*n* = 923)^a^	46/852 (5.4%)	5/71 (7.0%)	1.33 (0.51–3.45)
Stillbirth (*n* = 889) ^a^	14/822 (1.7%)	1/67 (1.5%)	0.87 (0.11–6.75)
Birth weight (g) (*n* = 887) ^a^	3069 (2640–3402)	2960 (2382–3420)	1.00 (0.99–1.00)
SGA^a^ (*n* = 860)	114/795 (14.3%)	12/65 (18.4%)	1.50 (0.77–2.92)
LGA^a^ (*n* = 860)	41/795 (5.1%)	8/65 (12.3%)	**2.78 (1.23–6.27)**
Macrosomia (birthweight > 4000 g)^a^ (*n* = 887)	20/822 (2.4%)	5/65 (7.7%)	**3.34 (1.21–9.22)**
5-min Apgar < 7^a^ (*n* = 904)	64/839 (7.6%)	3/65 (4.6%)	0.59 (0.18–1.92)

*OGTT* Oral glucose tolerance test, *GDM* Gestational diabetes mellitus, *DIP* Diabetes in pregnancy, *BMI* Body mass index, *DM* Diabetes mellitus, *PCOS* Polycystic ovary syndrome, *SGA* Small for gestational age, *LGA* Large for gestational age, *IQR* Interquartile range, *CI* Confidence interval

^a^Due to twin pregnancies, there were 1100 fetuses; the denominator refers to the number of fetuses/neonates

In the DIP group, patients delivered earlier than those in the GDM group ([weeks+days] 37 + 1 vs 38 + 2, OR 0.98, 95% CI 0.97–1.00), and delivery by cesarean section was more frequent (80.3% vs. 63.3%, OR 2.37, 95% CI 1.24–4.52). Moreover, the patients in the DIP group delivered large-for-gestational-age (LGA) and macrosomic newborns more often than the GDM group (12.3% vs. 5.1%, OR 2.78, 95% CI 1.23–6.27; 7.7% vs. 2.4%, OR 3.34, 95% CI 1.21–9.22, respectively). No other statistically significant differences were observed in other birth or neonatal characteristics. We defined LGA as a birthweight greater than the 90th percentile for the gestational age and small-for-gestational-age (SGA) as a birthweight less than the 10th percentile for the gestational age.

Regarding postpartum evaluation and screening, 522 patients returned for a follow-up appointment after parturition, corresponding to 49.06% of our cohort. There were no statistically significant differences between the groups in terms of the return rate for postpartum follow-up and screening. Approximately six-fold more patients in the DIP group had an abnormal postpartum OGTT. Table 2 summarizes the available postpartum screening data. Comparisons between patients who returned for postpartum evaluation and those who did not return for postpartum evaluation showed that only the age and usage of insulin at birth were statistically different, as seen in Table 3.

**Table 2:** Postpartum evaluation of women diagnosed with gestational diabetes

Variable (n of recorded data)	GDM Median (IQR) N (%)	DIP Median (IQR) N (%)	*Odds ratio* (95% CI)
Returned for postpartum OGTT	485/999 (48.5%)	37/65 (56.9%)	1.41 (0.85–2.33)
Abnormal OGTT	61/485 (12.6%)	17/37 (45.9%)	**5.91 (2.93–11.90)**
Fasting plasma glucose–OGTT (mmol/L) (*n* = 522)	4.7 (4.4–5.0)	5.0 (4.7–5.3)	**1.06 (1.03–1.09)**
2-h plasma glucose–OGTT (mmol/L) (*n* = 519)	5.6 (4.7–6.5)	6.8 (5.6–8.6)	**1.02 (1.01–1.03)**

*GDM* Gestational diabetes mellitus, *DIP* Diabetes in pregnancy, *OGTT* Oral glucose tolerance test, Three patients did not complete the test, *IQR* Interquartile range, *CI* Confidence interval

**Table 3:** Comparison between patients who returned and who did not return for postpartum evaluation

Variable (n of recorded data)	Returned for postpartum evaluation Median (IQR) N (%)	Did not return for postpartum evaluation Median (IQR) N (%)	*Odds ratio* (95% CI)
Age (*n* = 1064)	34 (30–38)	32 (27–37)	**1.05 (1.03–1.07)**
BMI (*n* = 1042)	27.5 (24.3–31.5)	27.2 (23.5–31.9)	1.00 (0.98–1.02)
Nullipara (*n* = 1064)	204/522 (39.0%)	221/542 (40.8%)	0.93 (0.73–1.19)
Family history of DM (*n* = 1064)	293/522 (56.1%)	284/542 (52.4%)	1.16 (0.91–1.48)
Previous GDM (*n* = 643) (for non-nullipara patients)	41/322 (12.7%)	34/321 (10.6%)	1.23 (0.76–2.00)
Connective tissue disease (*n* = 1043)	24/511 (4.7%)	17/532 (3.2%)	1.49 (0.79–2.81)
Chronic hypertension (*n* = 1064)	119/522 (22.8%)	144/542 (26.6%)	0.82 (0.62–1.08)
Asthma (*n* = 865)	21/441 (4.8%)	25/424 (5.9%)	0.80 (0.44–1.45)
Smoking habit (*n* = 1057)	33/519 (6.4%)	33/538 (6.1%)	1.04 (0.63–1.71)
Twin pregnancy (*n* = 1064)	13/522 (2.5%)	23/542 (4.2%)	0.58 (0.29–1.15)
PCOS history (*n* = 1002)	41/493 (8.3%)	41/509 (8.1%)	1.04 (0.66–1.63)
Hyperglycemia classification = DIP (*n* = 1064)	37/522 (7.1%)	28/542 (5.2%)	1.40 (0.84–2.32)
Congenital malformation (*n* = 887)	19/419 (4.5%)	30/468 (6.4%)	0.69 (0.38–1.25)
Stillbirth (*n* = 1064)	7/522 (1.3%)	6/542 (1.1%)	1.21 (0.41–3.64)
Insulin use at birth (*n* = 1045)	100/521 (19.2%)	70/524 (13.4%)	**1.54 (1.10–2.15)**

*BMI* Body mass index, *DM* Diabetes mellitus, *GDM* Gestational diabetes mellitus, *PCOS* Polycystic ovary syndrome, *DIP* Diabetes in pregnancy

We then divided the 522 women into two groups according to their puerperal 75 g-OGTT results. The first group comprised 444 women with normal postpartum glucose levels, while the second group comprised 78 women with abnormal postpartum OGTT results. Women with abnormal postpartum OGTT results were older (35 vs. 34, OR 1.06, 95% CI 1.02–1.11) and more frequently classified as DIP (21.8% vs. 4.5%, OR 5.91, 95% CI 2.93–11.90). Their characteristics are compared in Table 4.

**Table 4:** Clinical characteristics of women who received postpartum follow-up in the Gestational Diabetes Unit

Variable (n of recorded data)	Normal postpartum OGTT Median (IQR) N (%)	Abnormal postpartum OGTT Median (IQR) N (%)	*Odds ratio* (95% CI)
Age, years (*n* = 522)	34 (30–38)	35 (32–39)	**1.06 (1.02–1.11)**
BMI, kg/m^2^ (*n* = 515)	27.5 (24.4–31.6)	27.5 (24.1–30.8)	0.99 (0.94–1.03)
Family history of DM (*n* = 522)	250/444 (56.3%)	43/78 (55.1%)	0.95 (0.59–1.55)
Previous GDM (*n* = 323)	31/278 (11.2%)	9/45 (20%)	1.99 (0.88–4.52)
Chronic hypertension (*n* = 522)	100/444 (22.5%)	19/78 (24.4%)	1.11 (0.63–1.95)
Connective tissue disease (*n* = 511)	20/433 (4.6%)	4/78 (5.1%)	1.12 (0.37–3.36)
PCOS (*n* = 493)	31/418 (7.4%)	10/75 (13.3%)	1.92 (0.90–4.10)
Hyperglycemia classification = DIP (*n* = 522)	20/444 (4.5%)	17/78 (21.8%)	**5.91 (2.93–11.90)**
Twin pregnancy (*n* = 522)	12/444 (2.7%)	1/78 (1.3%)	0.47 (0.06–3.65)

*OGTT* 75-g oral glucose tolerance test, *BMI* Body mass index, *DM* Diabetes mellitus, *GDM* Gestational diabetes mellitus, *PCOS* Polycystic ovary syndrome, *DIP* Diabetes in pregnancy, *IQR* Interquartile range, *CI* Confidence interval

**Fig. 1:**
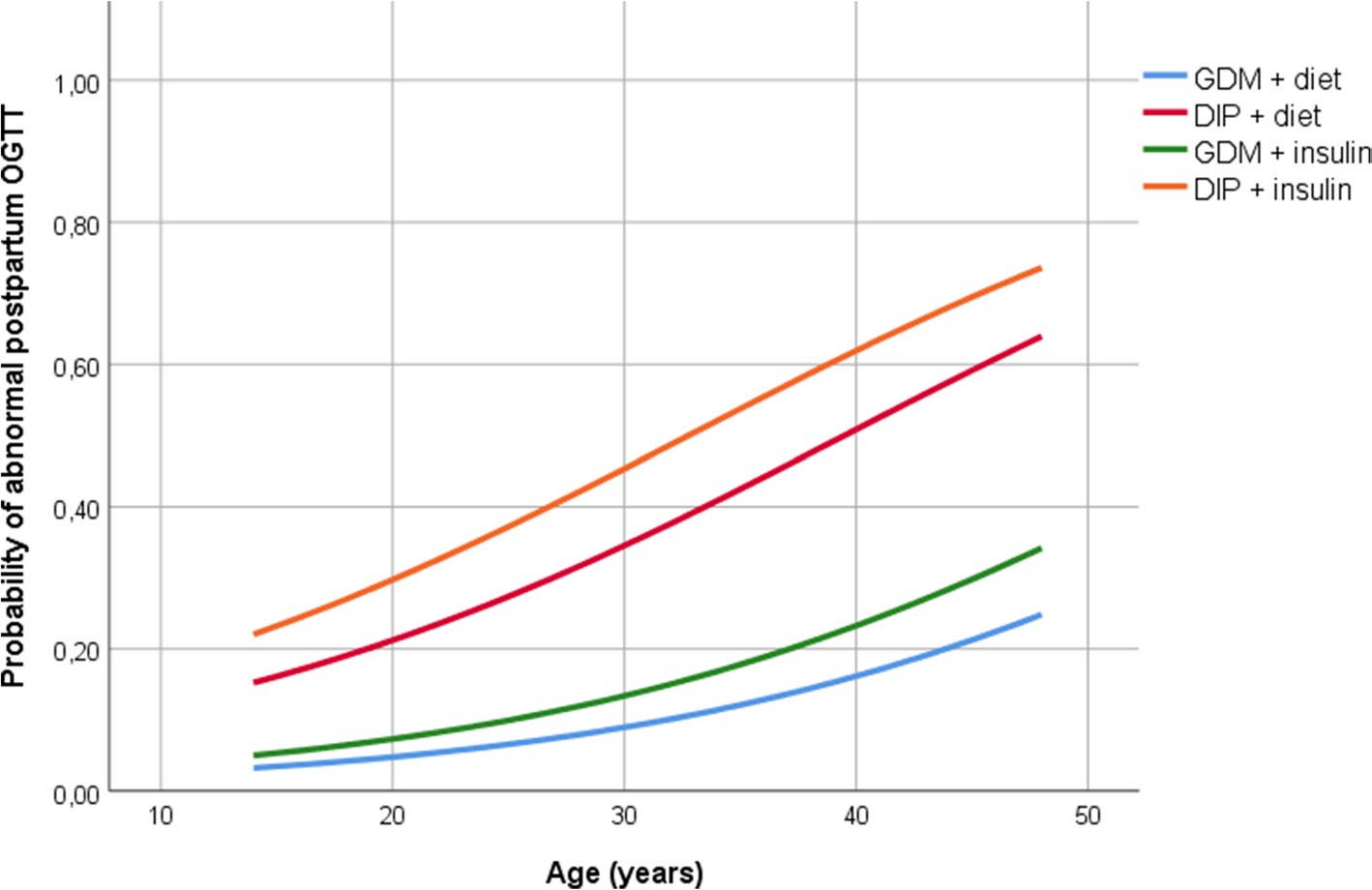
Probability of abnormal postpartum oral glucose tolerance test (OGTT) according to patient age and hyperglycemia classification during pregnancy (gestational diabetes mellitus [GDM] or diabetes in pregnancy [DIP]). The model also takes into account whether the patient’s blood sugar levels were managed by diet alone (diet) or whether insulin was required (+insulin). An abnormal result included diagnoses of impaired fasting glucose, impaired glucose tolerance, and diabetes mellitus

Multivariable logistic regression analysis was performed, including age and DIP, which were independent risk factors for an abnormal postpartum OGTT. Insulin use during pregnancy was also included because of its clinical relevance and because it was statistically significantly different between patients who did and did not return for the postpartum evaluation. The probability of having an abnormal postpartum OGTT was then calculated using the equation that is given below and graphically represented in Fig. 1.

$$p=\frac{\mathrm{Exp}\left[-4.79+\left(1.68, if\ DIP\right)+\left(0.067\ast age\right)+\right(0.453, if\ insulin\Big]}{1+\mathrm{Exp}\left[-4.79+\left(1.68, if\ DIP\right)+\left(0.067\ast age\right)+\right(0.453, if\ insulin\Big]}$$

## Discussion

In our comparison of women with DIP and those with GDM, the DIP group had more frequent histories of GDM, had higher pre-pregnancy BMIs, and were more likely to have chronic hypertension, current twin pregnancies, an insulin requirement, LGA infants, and abnormal postpartum OGTT results. Nevertheless, glucose levels normalized postpartum in more than half of these patients. This implies that meeting the WHO criteria for diabetes during pregnancy is associated with increased risk for some adverse perinatal outcomes but does not equate to a diagnosis of diabetes.

Pregnancy is a diabetogenic period characterized by hyperinsulinemia and increased insulin resistance, usually attributed to the effects of placenta-released hormones [2, 5]. Screening for hyperglycemia using the OGTT is typically performed late in the second gestational trimester when these pregnancy-related metabolic changes are more marked. Therefore, labeling a patient as diabetic because of an abnormal OGTT result during this period may be arguable.

The findings of higher BMIs, a more frequent history of GDM in a previous pregnancy, and a greater proportion of chronic hypertension in the DIP group agreed with previous reports that overweight/obesity, history of GDM, and hypertension are risk factors for elevated blood glucose [1].

Interestingly, more patients in the DIP group were carrying twins. The literature on GDM and multiple-pregnancies is inconclusive and even contradictory [6–8]. Of the 36 patients with twin pregnancies in this cohort, only 13 returned for the puerperal follow-up and screening. Only one of these, from the GDM group, had an abnormal puerperal OGTT result (IGT), confirming that pregnancy (and particularly multiple pregnancies) is associated with a metabolic challenge, and may not be a proper window for establishing a chronic diabetes diagnosis.

The rate of insulin use during pregnancy was significantly higher in the DIP group, with 46.1% requiring pharmacological treatment to achieve adequate glycemic control, compared to 14.3% of the GDM patients. The insulin-use frequency in our cohort was lower than that described by Sugiyama et al. [9] in a Japanese multicentric study. However, different criteria for GDM diagnosis were used in that study. As there are no globally accepted criteria, different boards and colleges suggest distinct tests and glucose thresholds for diagnosing GDM [3, 10–12], hampering comparison between studies. Other studies have suggested that 15–30% of women diagnosed with GDM cannot control glycemic levels with lifestyle modifications only and will require pharmacological treatment [3, 13].

Patients with DIP had more LGA and macrosomic newborns. Hyperglycemia in pregnancy is associated with an increased frequency of LGA, with an almost linear association between glucose levels and LGA newborns [14].

There was no significant difference between the groups in terms of the rates of infants who were SGA, congenital malformations, stillbirth and 5-minute Apgar score < 7. Congenital malformations are a well-established complication of pre-pregnancy diabetes, particularly in women with poor glycemic control during the peri-conceptional and fetal organogenesis periods [3, 10]. Thus, a higher incidence of fetal abnormalities in the DIP group might have been expected but was not observed. Our patients had normal first appointment FPG levels and therefore were not hyperglycemic during the peri-conceptional period. The high frequency of associated morbidity, particularly chronic hypertension, among our patients may explain the rates of SGA newborns above 10%.

Of the 522 patients with follow-up, only three were diagnosed with DM: one from the GDM and two from the DIP groups. In the GDM group, 12.6% had abnormal postpartum OGTT results (DM, IFG, and IGT), whereas, in the DIP group, the frequency was 45.9%. These numbers were lower than those described by Wong et al. [15] and comparable to those reported by Tovar et al. [16]. Importantly, although the DIP group had a six-fold higher rate of abnormal OGTT than the GDM group, more than half of the women in this group had normal OGTTs 6–12 weeks postpartum.

The strength of this study was the large number of cases reviewed and the fact that they were all treated as GDM, following the same protocol throughout the study, thus minimizing the effect of treatment protocols on the pregnancy outcomes. This study also had limitations, such as the relatively small number of patients who returned for postpartum evaluation (49.06%). Nevertheless, this rate is comparable to those described previously [17–19].

## Conclusions

Recognizing women with DIP and keeping them engaged in healthcare is necessary for closer monitoring during and post-pregnancy. This should include active puerperal follow-up, proper screening, and counseling. However, pregnant patients should not be labeled as diabetic based only on the glucose levels of the pregnancy OGTT, as this may inflict an unnecessary burden on the patients and the healthcare system.

Future studies should explore ways to increase the postpartum follow-up and screening rates and should prospectively study populations of women with hyperglycemia in pregnancy as well as women expecting twins.

## Data Availability

The datasets generated during and/or analyzed during the current study are available from the corresponding author on reasonable request.

## Abbreviations

GDM: Gestational diabetes mellitus
WHO: World Health Organization
IADSPG: International Association of Diabetes and Pregnancy Study Groups
FPG: Fasting plasma glucose
2 h-PG: 2-h plasma glucose
OGTT: Oral glucose tolerance test
DIP: Diabetes in pregnancy
IFG: Impaired fasting glucose
IGT: Impaired glucose tolerance
DM: Diabetes mellitus
BMI: Body mass index
PCOS: Polycystic ovary syndrome
OR: Odds ratios
CI: Confidence intervals
